# “Young Women My Age Really Need Boosts Like This”: Exploring Improv as a Facilitator of Wellness Among Young Women of Color

**DOI:** 10.1177/15248399221130726

**Published:** 2022-11-08

**Authors:** Stephanie Begun, Cam Bautista, Brigette Mayorga, Krysta Cooke

**Affiliations:** 1 University of Toronto, Toronto, Ontario, Canada

**Keywords:** child/adolescent health, health promotion, qualitative research, improv

## Abstract

This project qualitatively examined the potential of scenic improvisation (“improv”) for engaging young women of color as a possible means of promoting and enhancing health and wellness outcomes in this often-overlooked population. Seven young women of color (ages 15–18), accessing virtual after-school programming, participated in a three-session professionally facilitated improv workshop series. Participants provided insights through in-depth pre- and postproject qualitative interviews about their experiences. Participants indicated that improv boosted their self-esteem and strengthened their social connections. Participants were enthusiastic about accessing further improv opportunities, noting that improv should be embedded into other youth-serving programs and health-promotion efforts, as such approaches were deemed as particularly needed among young women of color. Inclusion of improv activities in intervention and prevention efforts would benefit from additional exploration as ways by which health and wellness programs and supports might be innovated and tailored to the specific needs and preferences of young women of color.

Scenic improvisation (“improv”) is the spontaneous production of unscripted responses to a scenario of the moment and involves impromptu acting, scene-development, and problem-solving. Circumventing needs for memorization, improv has shown to enhance focus, communication, compassion, and overall well-being (Bermant, 2013). Improv increases personal awareness, interpersonal attentiveness, and trust among group members, and is unavoidably social, whereby individual vulnerability contributes to collective strength (Bermant, 2013). Improv is a mechanism to generate laughter and humor, which reduces anxiety, stress, and loneliness, while improving energy level, feelings of empowerment, and restored hope (Berk, 2001). As an intervention, improv has demonstrated improvements in learning, sociability, self-esteem, coping, and help-seeking behaviors, as well as reduced mental health stigma (Morse et al., 2018; Wright et al., 2014).

Despite positive outcomes observed, gaps exist regarding improv’s potential to nurture creativity, self-esteem, and social connectedness among youth, generally, and more narrowly, young women of color. This group has been notably impacted by multiple pandemics, including social isolation and disproportionate familial and community losses due to COVID-19, coupled with state-sanctioned violence waged against communities of color. This project thus aimed to explore group-based improv participation experiences among young women of color.

## Evaluation Approach

Youth accessing virtual after-school programming designed for young women ages 14–18 in Scarborough, Ontario were recruited through program staff announcements and flyers circulated to youth via e-mail. A group of six to eight participants was sought as an “ideal” group size per (improv partner organization’s) recommendations. After youth expressed initial interest, informational interviews were conducted with youth via Zoom. Youth provided written informed consent to the interview and subsequent project participation if aged 16 and over; youth aged 14–15 were required to seek a guardian’s permission for the interview and subsequent participation. The University of Toronto Research Ethics Board approved the study procedures.

A structured interview guide aided in asking youth about their interests and hesitations in being involved in an improv project. Youth received CAD$25 honoraria for their interviews; youth were informed they would receive additional CAD$25 honoraria for each of the three workshop sessions and another for participating in a postproject interview (CAD$125 in incentives for 100% project participation). Nine young people expressed initial interest, with two opting not to be interviewed because the weekly workshop time did not align with their schedules. All seven young women who engaged in interviews chose to participate in the workshop series. Participants were aged 15–18; three identified as Asian and four as Black; all seven identified with the labels “young woman,” cisgender, and straight/heterosexual. Participants spoke English and had technology access for connecting to virtual improv sessions. Most participants were acquainted with one to two others from school or prior after-school programming, but none knew a majority of other participants at the start of this project.

Workshops met for 2 hours per week, via Zoom, for three sessions. The workshops were facilitated by an expert improv trainer from (partner organization) and co-hosted by the principal investigator (PI). Also present was a Masters-level Research Assistant, who kept informal jot-notes while participating in the improv activities. Each week, the group engaged in community-building, group norm-setting, and improv skill development, all of which were activities from (partner organization’s) improv curriculum. Participants were informed that they could turn off their cameras as they needed or wanted breaks, though they were encouraged to try to keep cameras on, as some activities engaged nonverbal elements. Each session concluded with a debriefing on how the activities were challenging, helpful, or relevant to daily life. Following the workshops, youth completed an interview, which again used a structured interview guide to learn about what youth liked, disliked, learned, and recommendations that arose from project-involvement.

Interview transcripts underwent qualitative template analysis by three coders, identifying data segments that correspond to a priori areas of inquiry and recognizing themes that emerge within such coding structures (Miles & Huberman, 1994). Codes that emerged within these a priori categories are reported, as follows.

## Lessons Learned

Prior to the workshops, the young women had little experience in improv. Some said that they were interested because improv sounded fun and presented an opportunity to meet new people and experience laughter, which was especially needed during COVID-19 lockdowns, online school, and times being difficult, in general. A few participants were hesitant, as their introverted natures made improv sound quite uncomfortable; yet, they indicated wanting to push themselves to try new things.

Following the workshops, youth shared resoundingly positive insights about their participation, noting that because of its interactive, group-based format, improv introduced meaningful connections to other young women and adult women facilitators, while also igniting self-confidence, self-esteem, and self-discovery of their own important voices and creativity. For these young women, improv proved meaningful as both a group-based activity for the social connections and teamwork that it required and inspired, as well as the personal insights that it facilitated within participants about their own personal gifts and strengths. Of note, participants willingly kept their cameras on much more than was predicted; despite their “Zoom fatigue,” they seemed to find that the collaboration and levity created through seeing and responding to others’ contributions to the group through scene and skit development made their visual involvement both necessary and fun. Most striking was that the young women who nearly did not participate due to introversion perhaps thrived most; one young woman recounted that her quiet, shy nature translated to excellent listening skills, a quality that is crucially important to improv collaboration and scene-building. She was noticeably inspired by her natural gifts for improv, and has actively stayed in touch with the facilitators, inquiring about ongoing opportunities for advanced improv training and education. Pre- and postproject qualitative themes and exemplar quotes are highlighted in Table 1.

**Table 1:** Qualitative Themes and Exemplar Quotes From Engaging Young Women of Color in Improv

Preproject insights from participants		
Question	Key theme	Exemplar quotes
What sounds potentially interesting or exciting to you about this group project?	Meeting new people	“It will be great to meet new people since we’re in lockdown forever”
	Sounds fun and different	“We need things for coming together that are not school. I like the sound of games and acting skills. It sounds like a fun break from the usual routine”
Is there anything that you are curious or concerned about, in terms of being a part of this brief improv project?	Introversion and nervousness	“I’m really introverted and I almost said no to doing this. I think it would be good for me. I can always not join in if I try it and don’t enjoy it. But I’m trying to get myself to do new things and put myself out there so it’s worth a try”
	Concerns about project being online	“I was concerned we had to turn on our cameras the whole time. I don’t love being on screen all the time, especially with school being online all day because it makes me tired, but this sort of activity is probably better with cameras on so it’s okay”
In the past, what types of things have you heard or experienced related to improv?	Little prior experience	“I did a play for drama class in grade 8 and although we had a script, we had to improv a little when someone messed up their lines. I don’t really know much about an actual improv program or class, though”
	Vague understanding of improv	“I’ve had to do lots of quick thinking, and coming up with words on the spot. I’ve heard you are never to say no, but continue the conversation. That’s about all I know”
What are you hoping to gain or learn from being a part of this experience?	Challenging oneself with new experiences	“I am hoping to learn how to be more comfortable putting myself out there and doing things I’m not necessarily good at”
	Laughter and enjoyment (amidst pandemic); stepping out of comfort zone	“I’m hoping to step out of my comfort zone a bit and honestly just have a good time. We could all use some laughs now especially with COVID and it seems like a good challenge for me personally”
What did you enjoy most about participating in improv?	Self-discovery of new strengths	“I really loved everything. I was able to explore traits that I never knew I had. I didn’t think this is something that I would love and as a quieter person, I didn’t think I would be very good at this, but I really surprised myself”
	Being oneself with other women	“It was great to be creative and positive and not worry about any of the actual outcomes—just being yourself and being creative with a group of other young women and women facilitators too”
*Postproject insights from participants*		
*Question*	*Key theme*	*Exemplar quotes*
What was most challenging for you about these improv activities?	Getting comfortable in beginning	“The initial push of getting comfortable with people I wasn’t close to, and putting myself out there—that was sort of a challenge in the beginning, but the way improv activities are, and how positive the facilitators are, that makes all of the ice melt and then you find yourself wanting to take risks and just jump in and try things. That was cool to experience”
	Hard to understand at first; online considerations	“Sometimes it took me a minute to understand the game or scene that were going to do, but after I understood things, it was really fun. I thought it would be hard to do this online but it actually wasn’t—it was kind of cool that way because we had a really watch each other for cues in a way that is more obvious when you’re in the same room”
What did you learn about yourself while participating in this project?	Increased willingness to participate	“That I’m not as bad as improv as I thought I would be. I found myself excited about my ideas and wanting to volunteer for things, and I’m not usually like that in a group activity. I usually like to hang back a bit but here I was ready to participate faster than what is typical for me”
	Creative self-discovery	“I am more creative than I realized. It takes some quick thinking and it’s easier to think quickly when you know there isn’t really a wrong answer.”
Do you see any ways by which improv could be helpful for people’s health or wellness?	Self-esteem and social connectedness	“Definitely self-esteem. And connection. I felt closer to the people in this group after just a few sessions—it’s like we said in one of our groups, you can’t leave anyone hanging. You do all of it as a real team”
	Confidence, finding one’s voice	“I found my own confidence growing each week and more like I have funny and important things to say so I should do so”
Thinking about the activities that we did together, are there any that stood out to you in some way?	Seeing different perspectives	“I really like the point of view game because it was very creative and gave a lot of dimension. It was great to see how people could think about the exact same scene in a million different ways”
	No wrong answers	“I liked how we did random word things at the beginning each time as ice-breakers. It was fun to know you could just blurt out any word and that it wouldn’t be wrong. That set a good tone for how we could do everything”
Would you recommend improv activities like this for others around your age?	Especially needed for young women	“I would definitely recommend this. I think it’s extra good for young women, as we struggle so much with our self-esteem and with being afraid to take risks, so this is a really, really great group to focus these activities and ideas on because girls and young women my age really need boosts like this”
	Brings people together, useful applications to daily life	“I think more young people should do improv activities like this. I think it really brings people together and there are useful lessons that are good for everyday life like just trying new things and surprising yourselves”

## Implications for Practice

Similar to prior research (Berk, 2001; Bermant, 2013; Morse et al., 2018; Wright et al., 2014), this project that engaged young women of color in group-based improv activities found promising support for improv as an approach to building social connections and fostering self-esteem. Participants enthusiastically reported that they would enjoy subsequent improv training opportunities, with several noting that though they were hesitant because of introversion and nervousness, they surprised themselves with their creative abilities. As improv endorses a “no wrong answers” and “yes and” approach to scene-building, participants must focus on being present, listening, and building with others in collaborative and nonjudgmental ways. Because improv calls upon participants to test internal boundaries by taking risks, along with its emphasis on showing external support for others’ contributions to the group, there may be utility in embedding improv activities into further dimensions of youth-serving programs. Though there were limitations (e.g., social desirability, incentives as motivation, small sample), the project was designed to be minimally invasive, with aims of creating comfortable, unique experiences for participants. Including improv activities in youth-serving, health-promotion programs would benefit from further exploration as ways of innovating and tailoring services to youths’ needs and preferences.
